# Re-do hypospadias surgery following failed previous repair: lessons learned over two decades of experience

**DOI:** 10.1007/s00383-024-05896-6

**Published:** 2024-11-16

**Authors:** May Fihman, Leon Chertin, Stanislav Kocherov, Jawdat Jaber, Boris Chertin, David Dothan

**Affiliations:** 1 Faculty of Medicine, The Hebrew University, Jerusalem, IL Israel; 2 The Department of Pediatric Urology Shaare Zedek Medical Center Faculty of Medicine, The Hebrew University, Jerusalem, IL Israel

**Keywords:** Hypospadias, Hypospadias surgery, Cripple hypospadias, Pediatric urology

## Abstract

**Purpose:**

To evaluate our experience with different surgical techniques and to find clinical factors that affect the outcome of treatment in cases of redo-hypospadias.

**Methods:**

We have retrospectively evaluated demographic and clinical data of children who underwent redo or cripple-hypospadias repair.

**Results:**

Between 2004 and 2021, 76 patients met the inclusion and exclusion criteria. The median age of the first cripple-hypospadias surgery was 64.8 ± 62.9 months. Upon primary surgery 5(6.6%) patients presented with distal-hypospadias, 13(17.1%) midshaft-hypospadias, 37(48.7%) proximal-hypospadias and 21(27.6%)with an unknown initial meatal status. To correct cripple-hypospadias 3(3.9%) patients underwent meatal-advancement and meatoplasty 32(42.1%) different tubularization techniques, 25(32.9%) required flap/graft, 13(17.1%) staged procedure and in 3(3.9%) surgical technique was undefined. Fifty-four (71%) children presented with post-surgery complications: 25(32.9%) meatal retraction, 19(25.3%) meatal stenosis and 17(22.3%) developed urethro-cutaneous fistula. Thirty-six (47.4%) patients underwent additional surgeries. There was no association between surgical technique or age and the need for additional surgeries (*P* = 0.831, *P* = 0.425 respectively). There was no association between surgical technique or age and surgical complications (*P* = 0.514, *P* = 0.425 respectively). All surgical techniques except meatal-advancement might lead to urethral stricture on long term follow-up (*P* = 0.028).

**Conclusions:**

Our data show that treatment of cripple-hypospadias is challenging for both surgeon and patients alike. There is a need to tailor a surgical technique to each patient and there is no one technique which is appropriate for all patients.

## Introduction

Cripple hypospadias is a term used frequently to describe a patient who underwent multiple surgical attempts to repair hypospadias. The consequence of the multiple surgical attempts is a penis with diminished blood supply, scarred skin and often residual chordee [1–3].

There is no consensus on the ideal treatment for the repair of cripple hypospadias. Many surgical techniques were previously suggested. The different techniques could be broadly grouped by single stage vs multiple stages, or those that require a graft or flap vs tubularization techniques utilizing local skin tissue. The goals of any surgical treatment are to form an esthetical looking penis, straight penis enabling sexual intercourse and a meatus at the tip of the penis that allows a straight urinary stream.

The major concern in this group of patients is the high complication rates and redo surgeries due to frequent complications such as: urethrocutaneous fistula, glans dehiscence, meatal stenosis, urethral stricture, diverticulum and recurrent ventral curvature [1, 3, 4]. It has been reported that complication rates requiring redo surgeries could be as high as 40% compared to 12% in patients undergoing primary hypospadias repair. Furthermore, the complication rates increase by 1.5 folds in each subsequent surgical attempt [5].

We have speculated that there is no unique technique or approach to manage all patients with hypospadias cripple and therefore have undertaken this study to compare the outcome of the different surgical techniques to correct cripple hypospadias in our department and to find variables that favor any specific surgical technique or predict repair failure.

## Methods

This is single center retrospective study. All procedures were in accordance with the ethical standards of the responsible committee on human experimentation (institutional and national) and with the Helsinki Declaration of 1975, as revised in 2000. The need for informed consent from all patients was waived by the IRB (institutional review board). De-identified data were used. Following permission from the IRB(0169–23-SZMC), we retrospectively collected demographic and clinical characteristics, complication rates and redo surgery rates from all children under the age of 18 years who underwent cripple hypospadias repair in the department of pediatric urology in our institute since 2004 until present. The majority of cases were performed by a single surgeon (B.C.) and a minority were performed by other pediatric urology surgeons (S.K., J.J.) with his supervision.

Inclusion criteria were: age under 18, clinical definition of "cripple hypospadias" in the medical records. We have defined cripple hypospadias as a patient who undergone surgical attempt to correct hypospadias and was left with scared tissue, bad cosmetic and functional outcome and combination of: abnormal meatus, urethral stricture, urethral dehiscence and fistulas [1, 3, 6]. We have excluded patients who developed: urethro-cutaneous fistula only and those who required glanuloplasty repair due to glans dehiscence.

For normally distributed continuous data, single comparisons were performed using Student’s t-test; for non-normally distributed continuous data, the Mann–Whitney U test was utilized. For categorical data, the chi-square test or Fisher’s exact test was used when appropriate (*n* < 5 in any field). A multiple logistic regression model was used to compare different surgical techniques. A two-sided *P* < 0.05 was considered statistically significant.

## Results

Between 1995 and 2021, 76 patients met the inclusion criteria. The demographic data is presented in Table 1. In brief, the median age at the first hypospadias surgery was 15.7 ± 14.8 months (Median ± SD). The median age of the first redo or cripple hypospadias surgery was 64.8 ± 62.9 months. Upon primary hypospadias surgery, 5(6.6%) patients presented with distal hypospadias, 13(17.1%) had midshaft hypospadias, 37(48.7%) with proximal hypospadias and in 21(27.6%) initial meatal status was undefined precisely. Since our pediatric urology department is a major referral center for challenging redo cases, some patients presented with cripple hypospadias even after their first surgical attempt by inexperienced hands 17(22.4%) or after a second attempt 30(39.5%).

**Table 1:** Demographic characteristics

Patient characteristics	Number	Percent
Number	76	
Median age (Median ± SD, months)		
First surgery	15.7 ± 14.8	
Cripple surgery	64.8 ± 62.9	
Meatal location		
Proximal	37	48.7%
Middle	13	17.1%
Distal	5	6.6%
Unknown	21	27.6%
Previous surgeries		
1	17	22.4%
2	30	39.5%
3	16	21.1%
4	10	13.2%
5	2	2.6%

We have combined the surgical techniques into four larger groups. The different techniques used in the primary hypospadias surgery and in the first cripple hypospadias surgery are presented in Table 2. In the primary hypospadias surgery 31 (40.8%) patients had chordee repair during the initial surgery. Of those, 6(19.3%) required only skin release, 7(22.6%) plication, 4(12.9%) patients required Nesbit procedure, 3(9.7%) patients required corporotomies and in 11(35.5%) chordee repair technique was not defined precisely. In the primary hypospadias surgery, 3(3.9%) patients underwent advancement techniques (MAGPI [100%]), 15(19.7%) underwent tubularization techniques (TIP 14[93%], Thiersch-Duplay 1[7%]), 20(26.3%) underwent procedure with flap or graft (Onlay pedicled island flap 12[60%], Tubularized pedicled island flap 2[10%] and Mathieu perimeatal-based flap repair 6[30%]) and the remaining 11(14.5%) underwent staged procedure ( Eight [73%] Preputial flap and in 3[27%] technique was not precisely defined). All staged procedures were comprised of two stages; the first stage included chordee repair and formation of urethral plate preferably from preputial pedicle flap as on lay, and when prepuce was not available mucosal graft was utilized. In the second stage tubularization was performed. Thirty-six (47.4%) patients had another urological comorbiditie such as: cryptorchidism or inguinal hernia. In 23(64%) it was corrected simultaneously during the hypospadias surgery.

**Table 2:** Surgery techniques in the primary and cripple hypospadias surgery

Surgery technique	1st hypospadias surgery	Cripple hypospadias surgery
Advancement	3	3
MAPGI	3	2
Anterior advancement		1
Tubularization	15	32
TIP [Snodgrass]	14	23
Thiersch Duplay	1	9
Staged procedure	11	
2 stages		13
Flap or Graft	20	25
Onlay pedicled island flap	12	1
Tubularized pedicled island flap	2	
Mathieu perimeatal-based flap repair	6	24
Unknown technique	27	3

To correct cripple hypospadias 28(36.8%) patients underwent residual chordee repair during the cripple surgery. Sixteen (57%) patients required skin release only, 7(25%) needed plication techniques, 2(7%) Nesbit technique, 1(3.6%) corporotomy and in 2(7%) patients chordee repair technique was unknown.

In the cripple hypospadias surgery three(3.9%) patients required advancement techniques (MAGPI 2[66%] and Anterior advancement 1[33%]), 32(42.1%) underwent tubularization techniques (23[72%] TIP repair and 9[28%] Thiersch-Duplay technique), 25(32.9%) procedures requiring flap or graft (24[96%] Mathieu perimeatal-based flap repair and 1[4%] Onlay pedicled island flap), 13(17.1%) staged procedure (4[30%] utilized Low Lip mucosal graft, 3[23%] Preputial pedicelled flap as onlay, 1[7%] Buccal mucosal graft, 5[38%] staged repair techniques were unknown) and in 3(3.9%) surgery technique was not precisely defined. The majority of the staged procedures included two stages (as described above), only one staged procedure was comprised of three stages (in the first stage only chordee repair is performed and the other two stages are identical to the two-stage technique described above).

Fifty-four (71%) children developed post hypospadias cripple surgery complications (Table 3): meatal retraction in 25(32.9%), meatal stenosis in 19(25.3%), urethra-cutaneous fistula in 17(22.3%), glans breakdown in 9(11.8%), urethral stricture in 5(6.6%) and 12 (15.8%) developed residual chordee.

**Table 3:** Complications after cripple hypospadias surgery

	Complication	*P* value
Metal stenosis	19 (25.3%)	0.811
Advancement	1 (33.3%)	
Tubularization	8 (25%)	
Staged procedure	2 (15.4%)	
Flap or Graft	8 (32%)	
Glans breakdown	9 (11.8%)	0.791
Advancement	0	
Tubularization	4 (12.5%)	
Staged procedure	1 (7.7%)	
Flap or Graft	4 (16%)	
Urethral stricture	5 (6.6%)	0.028
Advancement	0	
Tubularization	2 (6.3%)	
Staged procedure	1 (7.7%)	
Flap or Graft	2 (8%)	
Retraction of meatus	25 (32.9%)	0.811
Advancement	1 (33.3%)	
Tubularization	10 (31.2%)	
Staged procedure	3 (23.1%)	
Flap or Graft	11 (44%)	
Urethra-cutaneous fistula	17 (22.3%)	0.475
Advancement	0	
Tubularization	11 (34.4%)	
Staged procedure	2 (15.4%)	
Flap or Graft	4 (16%)	
Residual chordee	12 (15.8%)	0.339
Advancement	0	
Tubularization	6 (18.8%)	
Staged procedure	4 (30.8%)	
Flap or Graft	2 (8%)	

Thirty-six (47.4%) patients underwent additional surgeries: 11(14.5%) had meatoplasty, 10(13.2%) had fistula closure, 7(9.2%) underwent redo chordee release and 13(17.1%) underwent another hypospadias surgery repair (4 [30%] Mathieu perimeatal-based flap repair, 3[23%] Thiersch-Duplay technique, 2 [15%] Anterior advancement, 1[7.7%] MAPGI, 1[7.7%] TIP, 1[7.7%] Staged procedure, 1[7.7%] Onlay pedicled island flap). Fourteen patients with coronal meatus after the initial cripple surgery were satisfied and did not opt for subsequent surgeries.

With regards to surgical techniques, advancement techniques had 1(33.3%) complication, tubularization techniques 24(75%), techniques utilizing flap/graft 20(80%) and staged procedures 8(61.5, *P* = 0.3). One (33.3%) of the advancement techniques needed additional surgeries. In the tubularization techniques 18(56.3%) patients needed additional surgeries. In the techniques utilizing flap/graft 10(40%) and in the staged procedures 5(38.5%), *P* = 0.55.

We did not find any association between surgical technique or age at surgery and the need for additional surgeries (*P* = 0.549, *P* = 0.154 respectively) (Table 4). There was no association between surgical technique or age at surgery and surgical complications (*P* = 0.303, *P* = 0.425 respectively). Furthermore, additional urological procedures such as: orchidopexy, inguinal hernia repair, penoscrotal webbing repair… ext., did not significantly correlate with the need for more surgeries or post-surgery complications (*P* = 0.981, *P* = 0.22). Patients who have required residual chordee repair at the time of cripple hypospadias surgery did not have more complications or need for anymore surgeries (*P* = 0.956, *P* = 0.12). There was no association between the severity of hypospadias and post cripple surgery complications (*P* = 0.09): 29(78.4%) patients with proximal hypospadias, 7(53.8%) patients with midshaft hypospadias and 5(100%) patients with distal hypospadias had complications respectively.

**Table 4:** Association between different variables and the need for additional surgeries

	Any more surgeries	*P* value
Age (Mean ± SD, months)	80.95 ± 55.11	0.154
Meatal location	25 (45.5%)	0.325
Proximal	16 (43.2%)	
Middle	5 (38.5%)	
Distal	4 (80%)	
Surgical technique	35 (47.9%)	0.549
Advancement	1 (33.3%)	
Tubularization	18 (56.3%)	
Staged procedure	5 (38.5%)	
Flap/ Graft	10 (40%)	
Chordee repair	10 (35.7%)	0.120
Additional urological problems	17 (47.2%)	0.981

We have found a statistical correlation (*P* = 0.028) between the surgical technique and urethral stricture development. (Table 5). Two patients(6.89%) who underwent Thiersch-Duplay hypospadias repair, flap or graft 2(8.69%) (Mathieu perimeatal-based flap repair and Onlay pedicled island flap) and 1(7.69%) staged procedure (Buccal mucosal graft) developed urethral stricture respectively. None from the advancement surgery group presented with urethral stricture. Of those patients with urethral stricture: 3(60%) have had bulbar urethral stricture, 1(20%) penile and in 1(20%) localization of stricture was not known.

**Table 5:** Association between different variables and surgical complication

	Complications post-surgery	*P* value
Age ( Mean ± SD, months)	88.11 ± 62.85	0.425
Meatal location	41 (74.5%)	0.090
Proximal	29 (78.4%)	
Middle	7 (53.8%)	
Distal	5 (100%)	
Surgical technique	53 (72.6%)	0.303
Advancement	1 (33.3%)	
Tubularization	24 (75%)	
Staged procedure	8 (61.5%)	
Flap/ Graft	20 (80%)	
Chordee repair	20 (71.4%)	0.956
Additional urological problems	28 (77.8%)	0.220

## Discussion

Although there are more than 300 surgical techniques to repair hypospadias, cripple hypospadias remains one of the biggest challenges in pediatric urology [7, 8].

Some authors suggested that their method is superior in this type of patients [6].

Since we have vast experience in hypospadias repair, we have conducted a retrospective study to evaluate our experience and try to find if there is a surgical technique which is superior to others in terms of recurrence, complication rates etc. In addition, we sought-after any clinical variables which could predict cripple hypospadias repair failure.

Snodgrass et al. described that reoperative urethroplasty versus primary repair had doubled the complications rates already in the second attempt, and after the third re-operation there is 40% risk for complications [5]. This data emphasize the severity of the problem. Though the authors did not use the term “cripple hypospadias” it can be assumed that most patients who needed multiple surgical attempts to correct hypospadias have cripple hypospadias [5]. Our study, further iron out the challenges of redo/cripple hypospadias surgery. Seventy one percent of children in our study presented with post cripple hypospadias surgery complications during follow up. The most frequent complication was meatal retraction (32.9%), followed by meatal stenosis (25.3%), urethra-cutaneous fistula (22.3%), glans breakdown (11.8%), urethral stricture (6.6%) and residual chordee (15.8%).

Tamer et al. examined in a prospective trial the results of three types of cripple hypospadias repair: TIP, Thiersch-Duplay and Buccal mucosal graft [9]. They observed 20% complication rates in the first group, 13.3% in the second and 33.3% in the third. However, they did not perform a statistical analysis comparing the results of the three groups therefore we could not draw any meaningful conclusions with regards to the preferable technique in this clinical setup. In our study we evaluated numerous surgical techniques, however for the sake of reliable statistical analysis we have combined the surgical techniques into four larger groups: advancement techniques, tubularization techniques, single procedure with flap or graft and staged procedure. Despite the development of “hypospadiology” field we have not ultimately employed every new technique or approach in our services, but rather have preferred to tailor every operation to the individual case. Our data further demonstrate that there is no association between surgical technique and surgical complications or the need for additional surgery. We have found no difference between old school hypospadias surgery and new recently popularized techniques such staged approach, use of oral mucosa and etc.

Our experience particularly in staged repair further irons out aforementioned observations. In recent decades many pediatric urologists have suggested staged repair for complicated hypospadias including cripple hypospadias surgery [7, 10, 11]. This hypothesis is based on the fact that in staged repair a well vascularized graft is used for the reconstruction and does not relay on the damaged and scarred cripple hypospadias penile tissue. In our study 13(17.1%) of cripple hypospadias surgeries were staged. However, the fact that there was no difference between the postoperative complication rates or the need for additional surgeries between the different surgical techniques including the staged surgery group, we could not confirm that staged repair is superior to other techniques in the cripple hypospadias setting.

We did not find any association between the severity of hypospadias and post operative complications, although we expected that more proximal hypospadias will have significantly more complications since more proximal hypospadias frequently is accompanied by more severe curvature and hypoplastic urethral plate. We attribute this result to the “Law of small numbers”, since we had only a small number of patients in the midshaft and distal hypospadias groups [12]. Notably, there are 21(27.6%) patients for whom the initial meatal location is unknown. This missing data is due to our pediatric urology department being a major referral center, resulting in limited information about the initial meatal location, which prevents us from accurately defining it. In the Snodgrass study proximal/mid hypospadias patients with prior surgery had increased odds for complications (OR 1.63, 95% CI 1.01–2.63). He evaluated 1552 patients who underwent hypospadias surgery while 371 had re-operations ranging from 1 to 21 prior surgeries [5]. Similar outcome was found in Granier’s et al. study: 59.4% complication vs 34.2%, (*P* = 0.003) for scrotal hypospadias versus glandular\penile hypospadias. In her study 501 patients who underwent primary hypospadias surgery were evaluated [13].

In our study 14 patients were left with coronal meatus. Their original meatal location was perineal in 2(14%), penoscrotal 7(50%), midshaft 1(7%), coronal 1(7%) and 3(21%) unknown. These patients were satisfied with the surgical outcome. They were not bothered by the esthetical appearance of the coronal meatus nor had any functional bother such as deviated urinary stream or spraying. Sometimes the surgeon desires to put the meatus on the top of the glans which by any means might lead to numerous morbidities such as glans dehiscence, urethral diverticulum or dysfunctional voiding associated with hypoplastic penile glans in some patients with severe hypospadias, metal stenosis and narrowing glandular urethra. As we have shown the majority of these patients are doing very well with the coronary position of the meatus and not willing to undergo any further corrections.

We have found a correlation (*P* = 0.028) between the surgical technique and urethral stricture complication rate post-surgery. The majority of the strictures were in the bulbar urethra. Although the number of patients suffering from urethral stricture post-surgery is minimal, it is still noteworthy that in the flap or graft, staged procedure and tubularization techniques there were more recorded cases off urethral stricture while in the advancement surgery group none had developed this complication.

This study is not without limitations which worth to be mentioned. This is a retrospective study which suffers all flaws of a study of this kind. We have evaluated a relatively small number of participants; however, the diversity of the surgical techniques allows to perform a statistical evaluation. Only 38 patients completed follow-up through the adolescent period and their performance should be further evaluated. We did not use an objective questionnaire regarding cosmetic appearance of the penis following surgery. However, 46(60%) of our patients are mature enough to express their own satisfaction with the cosmetic and functional outcome of the surgery. Finally, we do not have data on sexual function in patients who have sexual experience. Functional outcome of the surgery in this group of patients will be an aim of our next study.

In conclusion, our data show that treatment of cripple hypospadias is challenging for the surgeon and patients alike. There is a need to tailor a surgical technique to each patient and there is no unique technique, which is appropriate for all patients.

## Data Availability

No datasets were generated or analysed during the current study.
